# Reply: HHV-8 transmission via saliva to soothe blood-sucking arthropod bites

**DOI:** 10.1038/sj.bjc.6602087

**Published:** 2004-08-10

**Authors:** J M Wojcicki

**Affiliations:** 1 Center for AIDS Prevention Studies (CAPS), University of California, San Francisco (UCSF) 74 New Montgomery Street, Ste 600, Box 0886, San Francisco, CA 94105, USA

**Sir,**

I thank M Coluzzi *et al* for their interest in my letter published in 2003 on traditional behavioural practices and exchange of saliva among sub-Saharan African populations. The comments by M Coluzzi *et al* on my letter highlight a potentially important behavioural practice associated with human herpesvirus 8 (HHV-8) transmission; the use of saliva to soothe blood-sucking arthropod bites. As mentioned in my letter, I found some evidence of this practice in reviewing ethnographic material from the Human Relations Area Files (HRAF). However, neither the extent nor frequency of saliva-associated behavioural practices, including those highlighted by Coluzzi *et al*, has been investigated among sub-Saharan African populations. The HRAF files present only descriptive, ethnographic data. Scientifically oriented epidemio-logical studies need to be developed to better characterise the frequency and distribution of saliva-associated behavioural practices and evaluate the potential association between these practices and risk of infection with HHV-8.

The ‘promoter arthropod’ hypothesis raised by Coluzzi *et al* (2002) concerning the use of saliva to soothe blood-sucking arthropod bites merits further investigation and may pertain to transmission of other viruses in addition to HHV-8. In their letter, Coluzzi *et al* discuss ecological data from a previous study Coluzzi *et al* (2003), suggesting a relationship between larviciding campaigns and decreased population HHV-8 seroprevalence in Sardinia. Although this ecological level data is intriguing, the association may be confounded by other time-dependent environmental or behavioural factors, and individual level studies are needed to test this hypothesis. In addition, in light of widespread infection with HHV-8 in diverse sub-Saharan African environments, ecological level analysis may be less informative in this context. Studies that assess the frequency of behavioural practices associated with saliva including treatment of insect bites and exposures to proposed environmental risk factors (e.g. the density of ‘promoter arthropods’) at the individual level are needed.
